# 

**Published:** 1889-12-14

**Authors:** 


### NOTICE TO CORRESPONDENTS.

All MS., Letters, Books for Review, and other matters intended for the Editor, should be addressed The Editor, The Lodge, Porchester Square, London, W.

### Notice to Advertisers and Subscribers.

All Advertisements, Orders for Copies of The Hospital, and Business Communications should be addressed to The Publisher, *not to the Editor*, The Hospital, 139-140, Salisbury-court, Fleet-street, E.C.

### Bound Volumes of "The Hospital."

Vol. VI., containing full page portraits of T.R.H. the Prince and Princess of Wales, and Miss Florence Nightingale, is now ready, 4s., post free. Cases for binding, may be had of the Publisher, at 1s. 6d. each.

### To Residents, Secretaries, and Matrons

The Editor will be glad to have the Regulations and each Report as ssued by Asylums, Hospitals, Convalescent and Nursing Institutions, as well as those of other Charities, and an early copy in duplicate of all Appeals and Festival Notices, etc., addressed to *The Editor, The Lodge, Porchester-square, London, W.* Any superintendent, secretary, matron, or other chief official who regularly sends such information may be registered, on application to the Publisher, to receive free of cost a cover for binding The Hospital in half-yearly volumes.

### Scraps and Gleanings.

Aberdeen proposes to establish a pay hospital.

Burton Infirmary Saturday collection has reached £805.

Glasgow Victoria Infirmary will be opened next month.

Dr. J. A. Macdonald has been elected physician to Taunton Hospital.

The Government of Victoria is about to establish a retreat for invalids.

Dr. Collyns has been appointed medical officer of Wimbledon Fever Hospital.

The National Children's Hospital, Dublin, held a successful bazaar last week.

The Duchess of Cleveland this week opened a bazaar at St. Leonards in aid of the local hospital.

Famine fever is raging in Moutiden, China, and missionaries are engaged in nursing the sick.

Mrs. Elliot and Mrs. Hall are about to publish the life of Laura Bridgman the celebrated deaf-mute.

A conference has been summoned for January 15th, at Birmingham, to consider the subject of hospital abuse.

Lady Herbert appeals for funds for the Westminster Soup Kitchen managed by the Sisters of Charity, of Carlisle-place.

The Moravian missionaries and nurses of the Leper Home at Jerusalem can show a perfectly healthy record as regards infection.

The result of the week of self-denial and prayer held by the Salvation Army in October last has, in round figures, amounted to £20,000.

Coombe Lying-in Hospital reports 857 in-patients and 1,819 out-patients treated during the last year. The hospital is in debt and needs funds.

At Blackpool a public meeting is to be held to further the establishment of a cottage hospital. A legacy of £1,000 was left in 1887 by Miss Lowe for this object.

Dr. John Thomas Banks, Physician in Ordinary to her Majesty in Ireland, has received the honour of knighthood, and was invested by her Majesty with the insignia of the Second Class Civil Division.

Gainsborough Hospital Saturday Committee have held their final meeting and presented their balance-sheet; £148 was received this year, being £10 more than last year. Working expenses were £14.

Mr. W. Anderson, F.R.C.S., Dr. Johnson Symington, and Dr. H. St. John Brooks have been nominated by the British board of selection as candidates for the Challis Chair of Anatomy in Sydney University.

Hospital-ity.—Hospital Physician (with a view to diagnosis): "What do you drink?" New Patient (cheering up at the proposal): "Oh, sir!—thank you, sir—whatever you——I leave that to you, sir!"—*Punch*.

Great Northern Central Hospital, N.—The number of patients relieved during the month of November was: medical, 1,943; surgical, 2,140; total, 4,083; of which 1,3[illegible]8 were new cases and 263 accidents.

"Death from pressure round the waist" was the verdict of a Birmingham jury at an enquiry into the death of a servant-girl, who received a severe fright. She was too tightly laced to stand any sudden emotion.

Under the patronage of his Royal Highness the Prince of Wales, a concert in aid of the Father Damien Fund will be given on Thursday afternoon, December 19, at 24, Park-lane, kindly lent by Lord Brassey for that purpose.

A despatch from Vienna shows that the Russian epidemic of influenza has reached the Austrian capital, where 13 cases are reported in the public hospital, chiefly young doctors. The epidemic preserves its mild character.

Sir Edward Guinness does not limit his benefactions to workmen's dwellings. He has given £1,000 to the Finsbury Polytechnic—a scheme for which the Duke of Westminster, Mr. Plunket, and Archdeacon Farrar are soliciting subscriptions.

Mr. James Henry Johnson, of the Abrain Coal Company, who gave as a Jubilee gift £5,000 to the endowment fund of the Wigan Mining and Mechanical School, has this week offered to build a children's wing for the Wigan Infirmary at a cost of £2,000.

A chemist's assistant in Magdeburg has been sentenced to a year's imprisonment for dispensing a morphia ointment instead of a calomel powder for a child, thereby causing its death. The assistant's principal was at the same time sentenced to three months' imprisonment.

Mr. C. Robins, F.S.A., delivered an address, "Reasons for the adoption of a uniform title of 'Sanitary Inspectors,' and suitable test examinations," at a meeting of the Association of Public Sanitary Inspectors, in the Carpenters' Hall, on Saturday evening, Mr. Hugh Alexander presiding.

The *Jewish Chronicle* contains a biography of Dr. Ali Cohen, of Groningen, who has just died. He was born at Meppel in 1817, and was one of the principal authors, with the statesman Thorbecke, of the modern Dutch hygienic legislation. In 1872 he founded the Netherlands society for cow-pox inoculation.

Under the title of "The Health of the People," Dr. Benjamin Ward Richardson will shortly publish, through Messrs. Longmans, an abridgment of "The Health of Nations"—a review of the works of Sir Edwin Chadwick—which appeared in two large volumes in 1887. The new work will be in one handy volume, containing a biographical introduction and portrait.

